# TNF-expressing CD1d^+^ monocytes are associated with the activation of CD4^-^ CD8^-^ T cells in patients with Chagas cardiomyopathy

**DOI:** 10.1590/0037-8682-0181-2024

**Published:** 2024-11-15

**Authors:** Carolina Cattoni Koh, Teresiama Velikkakam, Eula Graciele Amorim Neves, Nayara Ingrid Medeiros, Juliana Assis Gomes, Silvana de Araújo Silva, Kenneth John Gollob, Maria do Carmo Pereira Nunes, Walderez Ornelas Dutra

**Affiliations:** 1 Universidade Federal de Minas Gerais, Instituto de Ciências Biológicas, Belo Horizonte, MG, Brasil.; 2 Instituto Nacional de Ciência e Tecnologia em Doenças Tropicais, Salvador, BA, Brasil.; 3 Universidade Federal de Minas Gerais, Faculdade de Medicina, Belo Horizonte, MG, Brasil.; 4 Hospital Israelita Albert Einstein, São Paulo, SP, Brasil.

**Keywords:** CD1d^+^ monocytes, Chagas disease, Inflammation, Cardiomyopathy

## Abstract

**Background:**

Chagas disease cardiomyopathy is characterized by intense immune activation, with double-negative (DN) T cells as key producers of inflammatory cytokines. CD1d is an antigen-presenting molecule involved in the activation of DN T cells.

**Methods:**

We characterized CD1d^+^ monocytes from patients with cardiac (CARD) and indeterminate (IND) disease using flow cytometry.

**Results:**

CARD CD1d^+^ monocytes exhibited higher expression of TNF, TNF-receptor, PDL-1, and Fas-L compared to those from IND. These monocytes correlated with TNF expression by DN T-cells in CARD but not in IND.

**Conclusions:**

CD1d^+^ monocytes from CARD are inflammatory and associated with DN T-cell activation, confirming that CD1d is a target for modulating inflammation in Chagas cardiomyopathy.

Human infection with *Trypanosoma cruzi* leads to Chagas disease, in which most patients remain without cardiac alterations and are classified as indeterminate (IND), while approximately 30% develop severe cardiac manifestations (CARD)^1^. Despite the production of both pro- and anti-inflammatory cytokines, the immune environment in CARD patients is predominantly inflammatory^2^. Antigen-presenting cells expressing CD1 molecules primarily present glycolipid antigens to T cells, particularly CD4^-^ CD8^-^ (double-negative, DN) T cells^3^. These T cells express either alpha-beta (αβ) or gamma-delta (γδ) chains of the T-cell receptor, enabling them to identify pathogens and initiate an adaptive immune response^4^. DN T cells play a crucial role in CARD because of their prominent expression of cytotoxic markers (granzymes and perforin) and inflammatory cytokines (TNF and IFN-gamma)^5^ ^),(^ ^6^. Blocking DN T-cell activation by blocking CD1d-mediated antigen presentation results in a significant reduction in the inflammatory phenotype in CARD^7^ ^),(^ ^8^. This study aimed to characterize CD1d^+^ monocytes from IND and CARD before and after stimulation with *T. cruzi* antigens and evaluate the expression of activation molecules and cytokines, as well as their association with DN T-cell activation. 

This study included 22 Chagas disease patients with positive *T. cruzi* serology, divided into two groups: CARD (n = 12; 8 male, 4 female; average age ± SD: 59.81 ± 14.57) with heart failure symptoms, ventricular dilatation, global left ventricular dysfunction, and electrocardiographic abnormalities; and IND (n = 10; 8 male, 2 female; average age ± SD: 59.1 ± 12.91), asymptomatic, with normal clinical, radiological, and echocardiographic findings^1^. Ethical approval was obtained from the Comitê de Ética em Pesquisa of Universidade Federal de Minas Gerais and Comissão Nacional de Ética em Pesquisa (CONEP 2.809.859), and all volunteers provided informed consent.

Peripheral blood samples were collected in sodium heparin-containing sterile tubes. Peripheral blood mononuclear cells were obtained and cultured in medium alone (controls) or stimulated with *T. cruzi* antigen (20 μg/mL, from Y strain trypomastigotes), as previously described^8^. Following a 14-h incubation at 37°C in a 5% CO_2_ chamber, 1 μg/mL of Brefeldin A (BioLegend) was added for the last 4 h of culture. After incubation, cells were collected, washed, and immunostained for flow cytometry as routinely done by us^6^. Antibodies for phenotypic identification included anti-CD14 BV510 (clone 63D3), anti-Fas-L BV421 (clone NOK-1), anti-CD120a (TNF-R1) APC (clone W15099A), anti-CD1d PercpCy5 (clone 51.1), anti-HLA-DR APCcy7 (clone L243), anti-PD-L1 FITC (clone MIH2), anti-CD4 PercpCy5 (clone A161A1), anti-CD8 APCCy7 (clone SK1), anti-TCRab FITC (clone IP26), anti-TCRgd BV421 (clone B1), anti-TNF PE (clone Mab11), and anti-IL-10 PeCy7 (clone JES3-9D7). All antibodies were obtained from BioLegend. A minimum of 100,000 events from total lymphocytes and monocytes were acquired using a FACS Canto II flow cytometer (Becton Dickinson, San Jose, CA, USA). FlowJo software (Ashland, Oregon, US) was used for supervised data analysis, employing the doublet exclusion technique (FSC-A × FSC-H) and monocyte selection (FSC-A × SSC-A) (Figure 1A). Isotype controls and unstained cells were used to determine the negative staining for each examined fluorophore. Data were analyzed using GraphPad Prism® 8.0.2. The Shapiro-Wilk test was used to assess normality. Nonparametric data were analyzed using Wilcoxon tests for paired comparisons and Mann-Whitney tests for unpaired comparisons. Correlations were analyzed using the paired t-test, Pearson’s coefficient (parametric), and Spearman’s rank correlation (nonparametric). Linear regression was used to analyze treated correlation data. The significance level was set at P = 0.05. 

**FIGURE 1:**
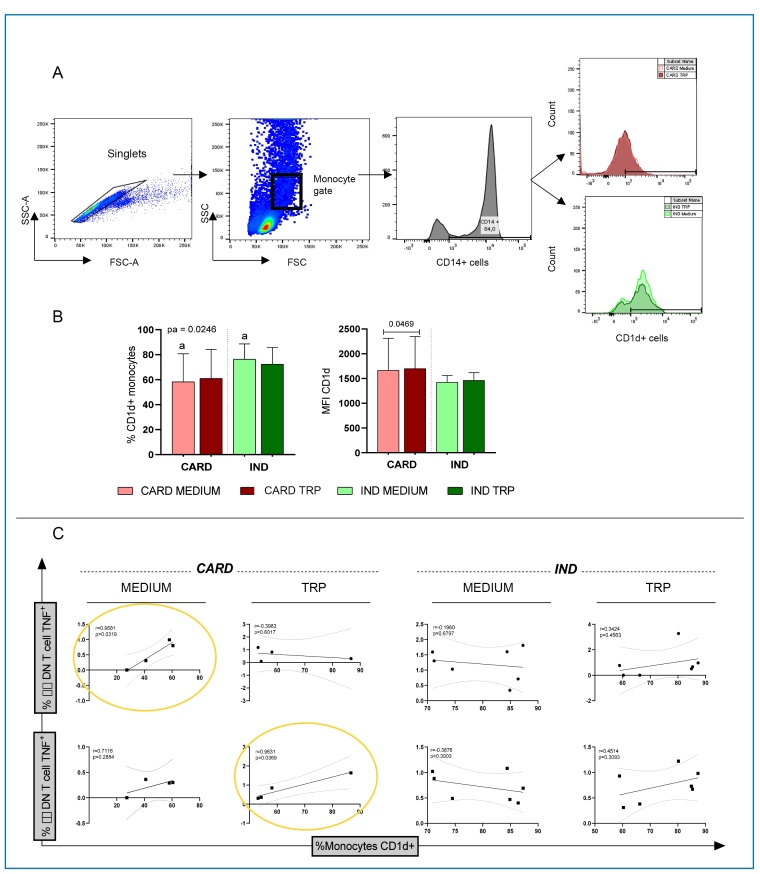
Percentage and intensity of CD1d expression in monocytes from patients with the cardiac (CARD) and indeterminate (IND) clinical forms of Chagas disease. The red bars represent the CARD group, and the green bars represent the IND group. Lighter shades indicate the medium, and darker shades represent *T. cruzi* antigen (TRP) stimulation. **(A)** Gating strategy for the analysis of CD1d^+^ cells and their functional characteristics, showing selection of singlets, monocyte population, CD14^+^ cells, and CD1d expression. **(B)** Frequency and mean intensity of expression of CD1d by monocytes from CARD and IND clinical forms in the absence (Medium) or presence of *T. cruzi* antigen stimulation (TRP). Results are presented as mean ± standard deviation. Identical letters or the horizontal bars represent statistically significant differences. Values of P<0.05 were considered statistically significant. **(C)** Correlation between the frequency of TNF^+^ expression in αβ double-negative T cells (first row) or γδ double-negative T cells (second row) and the frequency of CD1d^+^ monocytes. Statistical significance (P = 0.05) is indicated by yellow circles. "r" represents the correlation coefficient.

We initially evaluated the profile of the total circulating monocytes from IND and CARD by determining the expression of PD-L1, Fas-L, and IL-10 to assess their regulatory potential, and TNF and CD120a to evaluate their inflammatory profile^2^. Expression of a single marker was sufficient to indicate the potential regulatory or inflammatory profiles of monocytes. Table 1 shows that monocytes from CARD displayed a more inflammatory profile, with higher expression of TNF and CD120a compared to those from IND, especially after *T. cruzi* antigen stimulation (TRP). TRP increased the frequency of TNF^+^ CD120^+^ monocytes in both IND and CARD. Additionally, CARD monocytes displayed a high TNF/IL-10 ratio, corroborating their inflammatory characteristics. Fas-L expression was also elevated in CARD, and although TRP increased PDL-1 expression in monocytes from both IND and CARD, no differences were observed between IND and CARD. In contrast to TNF expression, IL-10 expression was higher in IND monocytes than in CARD monocytes. 

**TABLE 1:** Expression of surface molecules, cytokines, and TNF-receptor 1 (CD120a) in circulating monocytes from patients with different clinical forms of Chagas disease.

	% TNF^+^ cells	% IL-10^+^ cells	Ratio TNF/IL-10	%CD120a^+^ cells	%CD120a^+^TNF^+^ cells	% FAS-L^+^ cells	% PDL-1^+^ cells
**Indeterminate**							
Non-stimulated	0.41 ± 0.3	3.96 ± 2.1^c^	0.14 ± 0.1^f^	30.58 ± 18.3^h^	0.38 ± 0.3 ^j^	1.66 ± 0.8^l^	10.27 ± 10.1^n^
**Indeterminate**							
Trypomastigote antigen-stimulated	0.77 ± 0.5^a^	2.9 ± 1.8^d^	0.3 ± 0.2^g^	22.6 ± 17.6^h.i^	0.59 ± 0.5 ^j^	1.56 ± 0.5^m^	32.30 ± 18.9^n^
**Cardiac**							
Non-stimulated	0.49 ± 0.5^b^	0.83 ± 0.9^c.e^	1.12 ± 2^f^	32.0 ± 11.4	0.90 ± 1.0^k^	2.94 ± 1.3^l^	11.65 ± 7.3^o^
**Cardiac**							
Trypomastigote antigen-stimulated	4.54 ± 5.0^a.b^	1.3 ± 1.4^d.e^	1.72 ± 1.9^g^	36.37 ± 16.4^i^	5.97 ± 6.7^k^	8.34 ± 6.9^m^	49.01 ± 33.9^o^

Values are expressed as mean ± standard deviation. Matching letters (a-m) indicate statistically significant differences between groups.

Our data corroborate previous studies that demonstrated that monocytes from CARD display a more inflammatory profile, with high expression of TNF and IL-12 compared to those from IND^9^ ^),(^ ^10^. The present study showed that in addition to expressing more TNF, stimulation with *T. cruzi* antigen leads to higher expression of the TNF-receptor, potentially rendering CARD monocytes more responsive to this cytokine. This facilitates an autocrine response to TNF, which maintains the inflammatory and activated profile of these cells in CARD.

We then sought to evaluate the functional characteristics of CD1d^+^ monocytes in Chagas disease patients, given the importance of these cells in presenting glycoconjugate antigens, which are highly prevalent in the surface of *T. cruzi* ^11^. First, we determined the percentage and intensity of CD1d expression in monocytes from well-characterized IND and CARD after selecting the CD14^+^ population, as shown in the gating strategy presented in Figure 1A. We observed that while the frequency of CD1d expression in CD14^+^ monocytes was higher in the IND, the intensity of CD1d expression per cell was higher in monocytes from the CARD (Figure 1B), suggesting activation. The lower percentage of CD1d^+^ monocytes in the CARD than that in the IND may reflect the recruitment of these cells to the inflammatory infiltrate in the heart. However, in situ analyses is required to confirm this hypothesis. TRP stimulation did not alter the frequency of CD1d expression in monocytes from the IND and CARD groups; however, the intensity of expression was higher in CARD after stimulation (Figure 1B). We then evaluated whether the frequency of CD1d^+^ monocytes correlated with the activation of inflammatory DN T-cells, specifically αβ and γδ subsets, in IND and CARD. Our results showed that the frequency of CD1d^+^ monocytes was positively correlated with the frequency of DN T αβ cells expressing TNF in CARD, particularly in the absence of stimulation, while a significant correlation with DN T γδ cells expressing TNF was observed in the TRP-stimulated group. These correlations were observed in CARD but not in IND (Figure 1C). Given the association of CD1d^+^ monocytes with DN T-cells activation in CARD but not in IND, we investigated the characteristics of these monocytes in both CARD and IND. 

Analysis of HLA-DR expression in CD1d^+^ monocytes showed a higher intensity of expression of this activation molecule in IND than in CARD, both without stimulation and after TRP stimulation (Figure 2A). Interestingly, TRP stimulation decreased HLA-DR expression in CD1d^+^ monocytes from IND but did not alter its expression in CARD CD1d^+^ monocytes. Previous studies have shown that PD-1/PDL-1 expression regulates the suppressive activity of regulatory T cells in CARD^12^. Here, we showed that TRP induced PDL-1 expression in CD1d^+^ monocytes from both IND and CARD, with a higher expression in CARD than in IND after antigenic stimulation (Figure 2A). TRP stimulation did not alter Fas-L expression in CD1d^+^ monocytes from either IND or CARD, but CARD CD1d^+^ monocytes displayed higher expression of this molecule than those from IND (Figure 2A). Our study is the first to analyze Fas-L expression in monocytes from patients with Chagas disease. The expression of Fas and Fas-L by T cells has been associated with immune response regulation in both Chagas patients^13^ and experimental *T. cruzi* infection^14^. The expression of PD-1 and Fas-L by CD1d^+^ monocytes may contribute to the control of long-lasting Chagas cardiomyopathy. Regarding cytokines, expression of TNF was higher in CARD CD1d^+^ monocytes compared to those from IND, especially after TRP stimulation (Figure 2B), whereas the opposite was observed for IL-10 expression (Figure 2B). The TNF/IL-10 ratio, which reflects the inflammatory profile, was higher in CD1d^+^ monocytes from CARD than those from IND, both before and after *in vitro* stimulation (Figure 2C). This shows that the inflammatory profile observed in total monocytes from CARD is mirrored in the CD1d^+^ monocyte subpopulation. It has been shown that inflammatory monocytes display a prominent antigen presentation activity, critical for T-cell activation^15^. Our data showed a strong positive correlation between the frequency of CD1d^+^ monocytes and activated inflammatory DN T-cells in CARD but not in IND. This suggests that this monocyte subpopulation plays a critical role in contributing to the inflammatory milieu observed in patients with CARD by activating a major source of inflammatory cytokines in Chagas disease. Therefore, targeting CD1d^+^ monocytes may serve as a potential target for controlling Chagas disease cardiomyopathy. 

**FIGURE 2:**
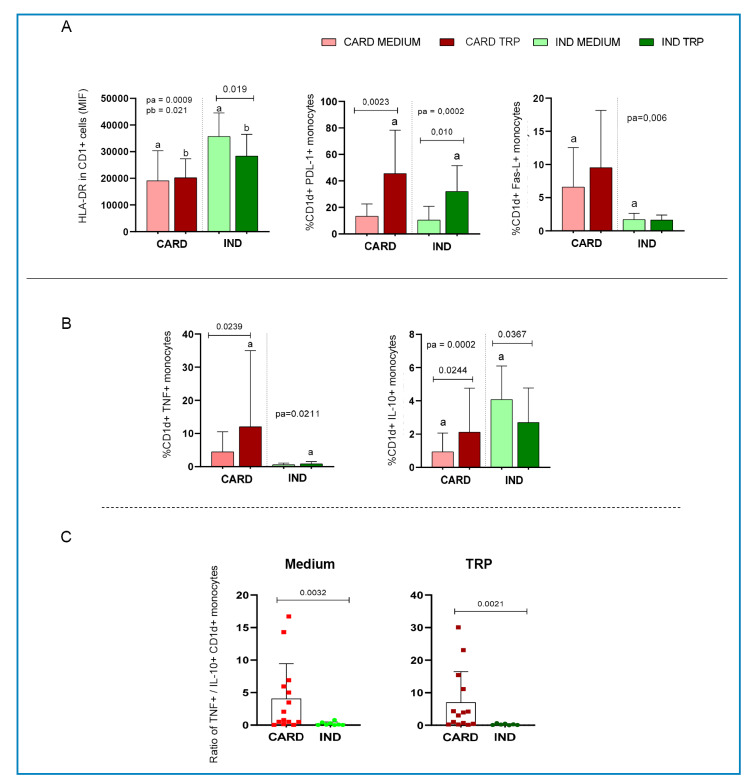
Analysis of expression of activation and modulatory molecules as well as cytokines by CD1d^+^ monocytes from patients with the cardiac (CARD)and indeterminate (IND) clinical forms of Chagas disease. The red bars represent the CARD group, and the green bars represent the IND group. Lighter shades indicate the medium, and darker shades represent *T. cruzi* antigen (TRP) stimulation. **(A)** Analysis of HLA-DR, PDL-1, and Fas-L expression by CD1d^+^ monocytes and **(B)** TNF and IL-10 by CD1d^+^ monocytes from CARD and IND patients, in the absence (Medium) or presence of *T. cruzi* antigen stimulation (TRP). Results are presented as mean ± standard deviation. Identical letters or the horizontal bars represent statistically significant differences. Values of P = 0.05 were considered statistically significant. (C) Ratio of TNF/IL-10 expression by CD1d^+^ monocytes from CARD (red) and IND (green) clinical forms in the absence (Medium) or presence of *T. cruzi* antigen stimulation (TRP). Graphs are presented as individual dispersion and mean ± standard deviation are demonstrated. P values are indicated in each graph.

## Data Availability

The corresponding author can provide data upon request.
